# Distinct Malignant Behaviors of Mouse Myogenic Tumors Induced by Different Oncogenetic Lesions

**DOI:** 10.3389/fonc.2015.00050

**Published:** 2015-02-24

**Authors:** Simone Hettmer, Roderick T. Bronson, Amy J. Wagers

**Affiliations:** ^1^Division of Pediatric Hematology and Oncology, Department of Pediatric and Adolescent Medicine, University Medical Center Freiburg, Freiburg, Germany; ^2^Department of Stem Cell and Regenerative Biology, Harvard Stem Cell Institute, Harvard University, Boston, MA, USA; ^3^Howard Hughes Medical Institute, Chevy Chase, MD, USA; ^4^Joslin Diabetes Center, Boston, MA, USA; ^5^Department of Biomedical Sciences, Tufts University Veterinary School, North Grafton, MA, USA

**Keywords:** rhabdomyosarcoma, myogenic differentiation, metastasis, transplantation

## Abstract

Rhabdomyosarcomas (RMS) are heterogeneous cancers with myogenic differentiation features. The cytogenetic and mutational aberrations in RMS are diverse. This study examined differences in the malignant behavior of two genetically distinct and disease-relevant mouse myogenic tumor models. *Kras; p1619^null^* myogenic tumors, initiated by expression of oncogenic *Kras* in *p16p19^null^* mouse satellite cells, were metastatic to the lungs of the majority of tumor-bearing animals and repopulated tumors in seven of nine secondary recipients. In contrast, *SmoM2* tumors, initiated by ubiquitous expression of a mutant Smoothened allele, did not metastasize and repopulated tumors in 2 of 18 recipients only. In summary, genetically distinct myogenic tumors in mice exhibit marked differences in malignant behavior.

## Introduction

Rhabdomyosarcomas (RMS) are heterogeneous cancers with myogenic differentiation (1). Fusion-positive RMS tumors carry exclusive chromosomal translocations at t(2;13)(q35;q14) or t(1;13)(p36;q14) and exhibit aggressive clinical behavior (2, 3). The remaining, fusion-negative spectrum of human RMS comprises a diverse group of tumors with frequent RAS pathway activation (4, 5) and variable mutations, including loss of heterozygosity at the *PTCH1* locus (6, 7) in a subset of fusion-negative RMS. PTCH1 serves as a Hedgehog (Hh) receptor, and loss of PTCH1 function results in de-repression of downstream Hh pathway signaling. The contributions of RMS-relevant oncogenic pathways, including RAS and Hh signaling, to myogenic tumor formation were previously tested in mice (8, 9). This report highlights the distinct phenotypes of two mouse myogenic tumor models – those initiated by combined *Cdkn2a* (*p16p19*) disruption and *Kras* expression in transplanted mouse muscle satellite cells (10) and those arising in the skeletal muscle of mice with activated Hh signaling due to expression of a mutant, constitutively active smoothened *(SmoM2)* allele (11, 12). We demonstrate significant differences in tumor-repopulating activity and prevalence of lung metastases between *Kras*-driven and Hh-driven myogenic tumors in mice. These observations reveal marked differences in malignant behavior between genetically distinct mouse myogenic tumors, suggesting that an understanding of the distinct oncogenetic underpinnings of tumors on the fusion-negative RMS spectrum may be informative for clinical prognosis and treatment.

## Materials and Methods

### Mice

*R26-SmoM2* (mixed genetic background including 129/Sv and Swiss Webster as main components) (11), CAGGS-CreER (11), and NOD.CB17-Prkdc^scid^/J (NOD.SCID) mice were purchased from The Jackson Laboratory. *p16p19^null^* mice (B6.129 background) were obtained from the NIH/Mouse Models of Human Cancer Consortium. Mice were bred and maintained at the Joslin Diabetes Center Animal Facility. All animal experiments were approved by the Joslin Diabetes Center Institutional Animal Care and Use Committee.

### Sarcoma induction

*Kras; p16p19^null^* myogenic tumors were initiated by fluorescence-activated cell sorting of *p16p19^null^* satellite cells, followed by lentiviral transduction to introduce oncogenic *Kras(G12v)* and implantation in the gastrocnemius muscles of NOD.SCID mice as previously described (10). *R26-SmoM2*;*CAGGS-CreER* were injected with Tamoxifen (1 mg/40 g) on postnatal day 10 to activate expression of CRE recombinase and SMOM2. *R26-SmoM2*;*CAGGS-CreER* spontaneously developed multifocal skeletal muscle tumors (*SmoM2* tumors) as previously described (11, 12).

### Histopathology

Tumor tissue was dissected, fixed in 4% paraformaldehyde for 2 h, and embedded in paraffin. Standard H&E stained sections were prepared. Staining for Actin (Dako, M0635, 1:200), Desmin (Dako, M0760, 1:50), and Ki67 Ki67 (Vector Labs, VP-K451, 1:250) was performed as previously described (10).

### Lung metastases

Tumor-bearing mice were monitored at least twice weekly for health problems, and were sacrificed once tumors reached a volume of 1 cm^3^ or were ill. Lungs were dissected, fixed in 4% paraformaldehyde for 2 h, and embedded in paraffin. Standard H&E stained sections were prepared and evaluated for the presence of metastases by Roderick T. Bronson.

### Tumor transplantation

Tumors were harvested, digested in DMEM + 0.2% collagenase type II (Invitrogen) + 0.05% dispase (Invitrogen) for 90 min at 37°C in a shaking waterbath, triturated to disrupt the remaining tumor pieces, and filtered through a 70 mm cell strainer. Red blood cells were lysed from tumor cell preparations by 3 min incubation in 0.15 M ammonium chloride, 0.01 M potassium bicarbonate solution on ice. Defined numbers of tumor cells were resuspended in 10–15 ml of HBSS with 2% FBS and injected into the gastrocnemius muscles of 1- to 3-month-old, anesthetized NOD.SCID mice using a transdermally inserted dental needle attached to a Hamilton syringe via polyethylene tubing. Recipient muscles were preinjured 24 h before cell implantation by injection of 25 ml of a 0.03 mg/ml solution of cardiotoxin (from *Naja mossambica*, Sigma) in order to enhance cell engraftment. Mice were screened once weekly for the development of tumors at the injection sites.

### Statistics

Differences between *Kras; p16p19^null^* and *SmoM2* mouse myogenic tumors were evaluated by *T*-test (Ki67 indices), Fisher’s Exact test (prevalence of lung metastases), and Kaplan–Meier analysis (tumor-repopulating activity).

## Results

### *Kras; p16p19^null^* and *SmoM2* mouse tumors exhibit a myogenic tumor phenotype

**Figure 1:**
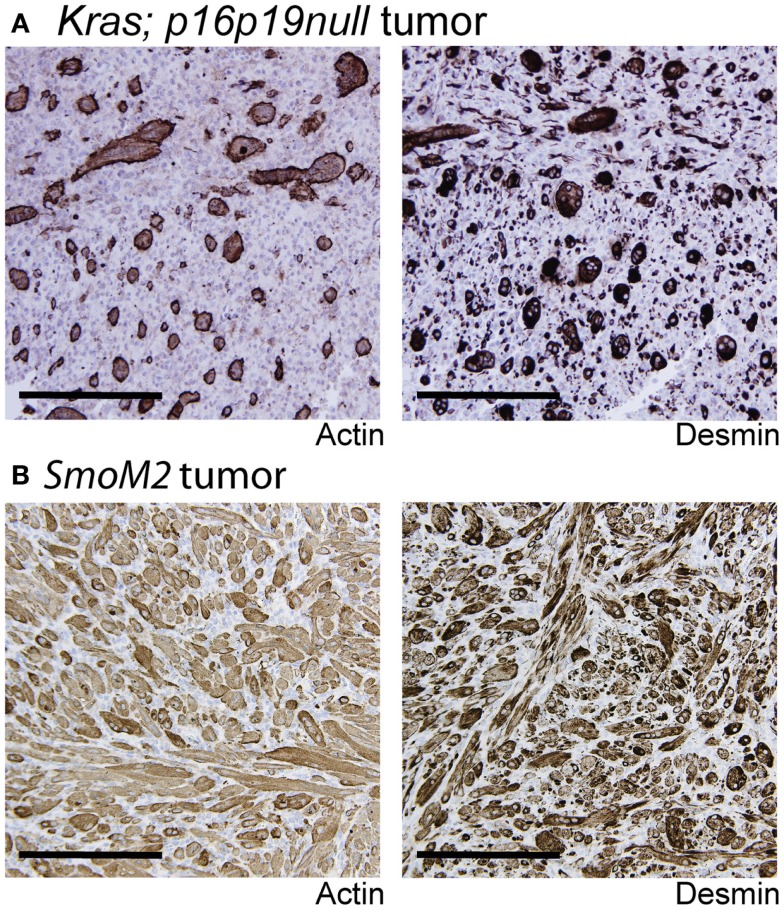
**Terminal myogenic differentiation in *Kras; p16p19^null^* and *SmoM2* mouse tumors**. **(A)** Subsets of *Kras; p16p19^null^* tumors cells express terminal muscle differentiation markers, actin and desmin. **(B)** The majority of SmoM2 tumor cells express actin and desmin. Images were taken at 20× (scale bars indicate 100 μm).

*Kras; p16p19^null^* mouse myogenic tumors were induced by intramuscular implantation of *Kras(G12v)*-expressing *p16p19^null^* muscle satellite cells (10). In contrast, SmoM2 mouse myogenic tumors were initiated by ubiquitous activation of a mutant, constitutively active smoothened *(SmoM2)* allele in *R26-SmoM2*;*CAGGS-CreER* mice (11, 12). The phenotypes of *Kras; p16p19^null^* and *SmoM2* myogenic tumors were previously described (10–12). In brief, *Kras; p16p19^null^* tumors contained bundles of cells with large, atypical nuclei, frequent mitotic figures, and occasional multinucleated giant cells. Subsets of cells (<50% of all tumor cells) expressed terminal muscle differentiation markers such as desmin and actin (Figure 1A), and the proliferative index as evidenced by the percentage of Ki67-expressing nuclei was 41.6 ± 12.5% (range 30.5–59.3%; four tumors evaluated) (Table 1). *SmoM2* tumors contained many multinucleated, elongated cells with abundant cytoplasm interspersed with small round cells. *SmoM2* tumors lacked cellular atypia and diffusely expressed desmin and actin in many tumor cells (more than 75% of all tumor cells; Figure 1B). As previously reported (12), the Ki67 index of *SmoM2* tumors was 19.1 ± 15.9% (range 3.4–41.8%; six tumors evaluated) and lower than that observed in *Kras; p16p19^null^* tumors (*p* = 0.05; Table 1).

**Table 1:** **Differences in the malignant behavior of *Kras; p16p19^null^* and *SmoM2* mouse tumors**.

	*Kras*; *p16p19^null^* tumors	*SmoM2* tumors
Terminal muscle differentiation	Actin/desmin expression in <50% of tumor cells	Actin/desmin expression in >75% of tumor cells
Ki67 index (*p* = 0.05)	41.6 ± 12.5%	19.1 ± 15.9%
Metastases (*p* = 0.001)	7 of 9 mice with lung metastases	0 of 10 mice with lung metastases
Transplantation (*p* < 0.001)	7 of 9 transplanted mice developed tumors (50 cells injected)	2 of 10 transplanted mice developed secondary tumors (100–150 k cells injected)

**Kras; p16p19^null^* and *SmoM2* mouse myogenic tumors exhibit profound differences in tumor-repopulating activity and metastatic behavior*.

### *Kras; p16p19^null^* and *SmoM2* mouse myogenic tumors have different metastatic potential

**Figure 2:**
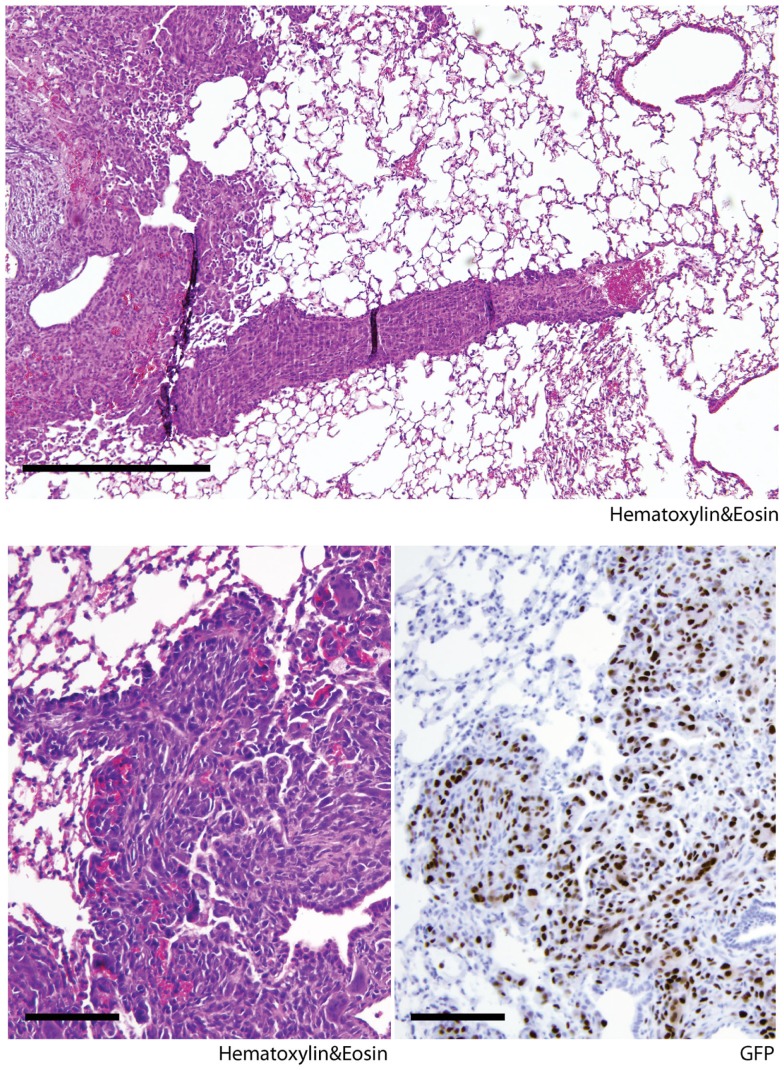
***Kras; p16p19^null^* mouse myogenic tumors metastasize to the lungs of tumor-bearing animals**. Random lung sections from *Kras; p16p19^null^* tumor-bearing mice show metastases. Tumor cells invade lung capillaries (top panel). Similar to primary tumors arising from GFP+ *Kras*-expressing; *p16p19^null^* satellite cells, lung metastases are GFP+ (bottom right panel). Images were taken at 10× and 20× (scale bars indicate 100 μm)

The lung is the primary organ affected by distant sarcoma metastases in humans. To assess the metastatic potential of *Kras; p16p19^null^* and *SmoM2* tumors, random lung sections obtained from tumor-bearing animals were screened for the presence of metastases. Six of seven mice with *Kras; p16p19^null^* myogenic tumors were found to have lung metastases at the time of death (mice were sacrificed 17–28 days after detection of palpable tumors) (Figure 2). In contrast, 0 of 8 mice with SmoM2 myogenic tumors had lung metastases at the time of death (mice were sacrificed at 38–55 days of age and 5–21 days after detection of palpable tumors). The prevalence of lung metastases in *Kras; p16p19^null^* and SmoM2 myogenic tumor-bearing mice was significantly different (*p* = 0.001).

### *Kras; p16p19^null^* and *SmoM2* mouse myogenic tumors differ in tumor-repopulating activity

**Figure 3:**
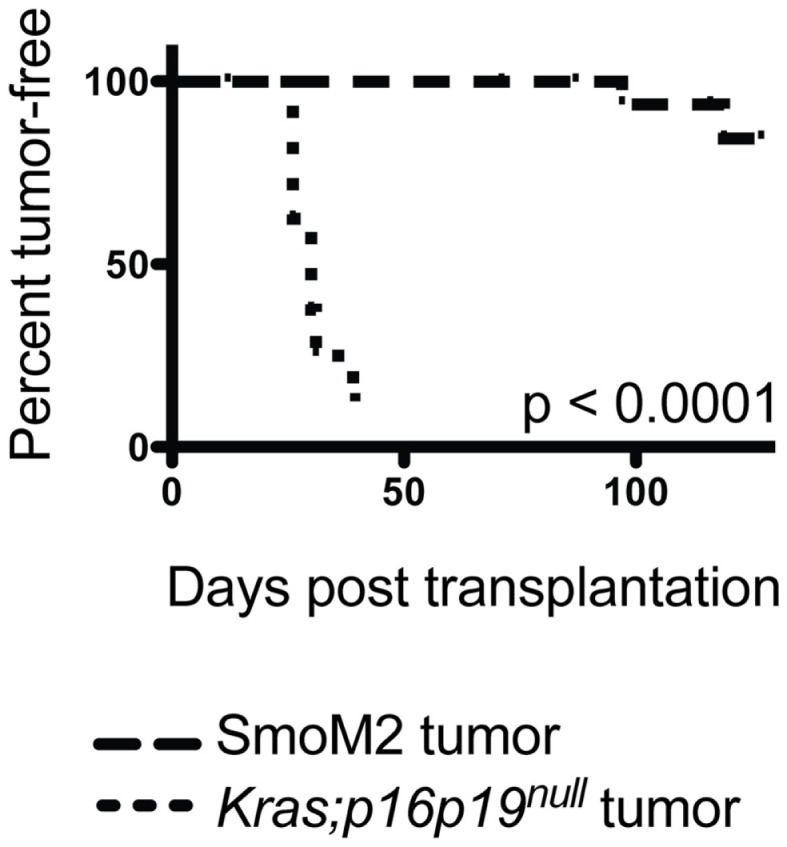
***Kras; p16p19^null^* tumor cells repopulate tumors in secondary recipients more effectively than *SmoM2* mouse tumor cells**. Pi^−^Ca^+^GFP^+^ *Kras; p16p19^null^* tumor cells were sorted independently from two primary tumors and injected into the cardiotoxin-pre-injured gastrocnemius muscles of NOD.SCID mice (50 cells per injection). Pi-Ca+ *SmoM2* tumor cells were sorted independently from four primary tumors and injected into the cardiotoxin-pre-injured gastrocnemius muscles of NOD.SCID mice (100,000–150,000 cells per injection). Recipient mice were monitored for the occurrence of secondary tumors at the injection site for up to 4 months.

Most malignant tumors contain cells that have the capacity to repopulate secondary tumors when transplanted into a susceptible secondary environment, and this assay has been used as a test of the malignancy of distinct tumors and tumor cell subsets (13). To evaluate the tumor-repopulating activity of *Kras; p16p19^null^* and *SmoM2* mouse myogenic tumors, viable tumor cells were transplanted into the cardiotoxin-pre-injured gastrocnemius muscles of NOD.SCID mice. The *Kras; p16p19^null^* tumor cell pool contains approximately 70% GFP+ cells and 30% GFP− cells (10). Because tumor-repopulating activity in *Kras; p16p19^null^* tumors resides within the Kras-expressing, GFP+ subset of tumor cells descended from virally infected satellite cells (Figure S1 in Supplementary Material), *Kras; p16p19^null^* tumor cells were sorted for transplantation from two *Kras; p16p19^null^* primary tumors as GFP+, Pi−, Calcein+ cells. Seven of nine mice injected with only 50 GFP+, Pi−, Calcein+ *Kras; p16p19^null^* tumor cells developed secondary tumors at the injection site 26–39 days after tumor cell injection. For *SmoM2* tumors, viable tumor cells were sorted as PI− Calcein+ cells from primary tumors obtained from four mice. Surprisingly, despite significantly higher numbers of cells transplanted (100,000 to 150,000 PI−, Calcein+ SmoM2 tumor cells per recipient), only 2 of 18 recipient mice developed secondary tumors, which were detected 71 and 127 days after cell injection. These experiments indicate marked differences in tumor-repopulating activity of *Kras; p16p19^null^* and *SmoM2* tumors (*p* < 0.001, Figure 3), in terms of both the frequency of tumor-repopulating cells and the latency of secondary tumor formation.

## Discussion

Our findings highlight differences in the malignant phenotype and behavior of mouse myogenic tumors driven by activation of distinct RMS-relevant oncogenic pathways. *Kras; p1619^null^* myogenic tumors were metastatic to the lungs of the majority of tumor-bearing animals and contained high tumor-repopulating activity. In contrast, *SmoM2* tumors did not metastasize and were substantially less effective in repopulating tumors in secondary recipients. These observations indicate that genetically distinct myogenic tumors in mice display marked differences in their malignant behavior.

The two model systems described in this study were induced by different experimental methods. *SmoM2* tumors originated from Cre-mediated activation of a conditionally expressed transgene. *Kras; p16p19^null^* mouse tumors, on the other hand, were initiated by viral transduction and intramuscular implantation of target satellite cells. We note that *Kras; Tp53*^−^*^/^*^−^mouse myogenic tumors (14, 15), induced by Cre-mediated activation of oncogenic hits instead of viral transduction, exhibit a phenotype that closely resembles the *Kras; p16p19^null^* mouse tumors described here. For example, *Kras; p16p19^null^* share their propensity to metastasize to the lungs of tumor-bearing animals with *Kras; Tp53*^−^*^/^*^−^mouse tumors (14). Nevertheless, it is possible that differences in the tumor induction strategy (such as off-target effects of viral transduction) could contribute to the observed differences in malignant behavior between *SmoM2* and *Kras; p16p19^null^* mouse myogenic tumors.

Similar to mouse myogenic tumors, human fusion-negative RMS comprises a group of tumors with clear differences in histology, myogenic differentiation state, oncogenic pathway activation, and genetic background. In recent years, subsets of human RMS tumors that exhibit a combination of specific genetic and phenotypic characteristics were distinguished. For example, a subset of human fusion-negative RMS with spindle cell/sclerosing histology was recently found to exhibit diffuse MyoD expression, carry frequent somatic MyoD mutations, and portend a poor prognosis (16, 17). Also, children with *TP53* germline mutations are predisposed to develop anaplastic RMS at a young age (18), and germline mutations in *DICER1* were linked to a genetic susceptibility to develop RMS of the genitourinary tract (19). Future extended (epi-)genotype/phenotype correlations might pinpoint clinically/biologically distinct subgroups of human fusion-negative RMS and identify biomarkers to facilitate prognostication and/or stratification of therapy.

## Author Contributions

SH, RB, and AW conceived experiments, analyzed data, wrote, and approved of the manuscript.

## Conflict of Interest Statement

The authors declare that the research was conducted in the absence of any commercial or financial relationships that could be construed as a potential conflict of interest.

## Supplementary Material

The Supplementary Material for this article can be found online at http://www.frontiersin.org/Journal/10.3389/fonc.2015.00050/abstract
