# Supplementary material for: Distinct Malignant Behaviors of Mouse Myogenic Tumors Induced by Different Oncogenetic Lesions
Source: Front Oncol. 2015 Feb 24;5:50. doi: 10.3389/fonc.2015.00050 (PMC4338657; doi:10.3389/fonc.2015.00050)
Supplement: Supplementary file 1 [file Image_1.PDF]

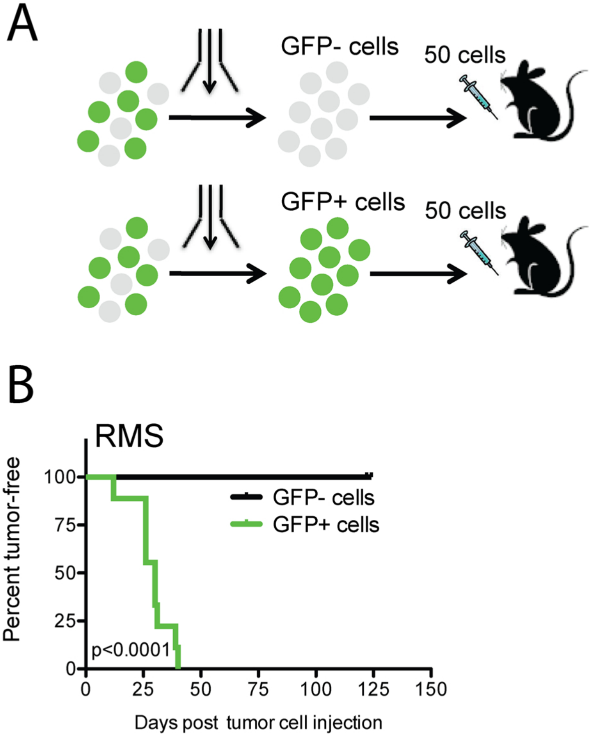

Figure S1. Tumor-propagating capacity in Kras;p16p19null RMS. (A) GFP-negative (top) or GFP-positive (bottom) tumor cells from primary RMS tumors (Hettmer et al., 2011) were isolated by FACS and transplanted into pre-injured secondary NOD.SCID recipients . Transplanted mice were monitored for tumor formation for up to 125 days. (B) Tumor-forming potential was restricted to the Kras-expressing, GFP-positive subset of cells ($p<0.0001$).
